# Qualitative inquiry with primary care providers and specialists about adult weight management care and referrals

**DOI:** 10.1093/tbm/ibac006

**Published:** 2022-02-23

**Authors:** Lisa Bailey-Davis, Angela Marinilli Pinto, David J Hanna, Chad D Rethorst, Christopher D Still, Gary D Foster

**Affiliations:** 1 Department of Population Health Sciences, Geisinger, Danville, PA 17822, USA; 2 Obesity Research Institute, Geisinger, Danville, PA 17822, USA; 3 Department of Psychology, CUNY Baruch College, New York, NY 10010, USA; 4 WW International, Inc., New York, NY 10010, USA; 5 Perelman School of Medicine, University of Pennsylvania, Philadelphia, PA 19104, USA

**Keywords:** Obesity, Counseling, Practice guidelines, Weight-loss treatment, Referrals

## Abstract

Obesity is a highly prevalent disease and providers are expected to offer or refer patients for weight management yet increasingly fewer clinical visits address obesity. Challenges to offering care are known but less is known about referrals and how specialists who treat obesity-related comorbidities address care and referrals. This study explored perceptions of primary care providers (PCPs) and specialty providers regarding care and referrals for weight management, specifically referrals to programs in the community setting. A qualitative design was used to interview 33 PCPs (mean age 54 years) and 31 specialists (cardiology, gynecology, endocrinology, and orthopedics [mean age 62 years]) in the USA during 2019. Each interview was conducted by telephone, audio-recorded, and transcribed verbatim. Inductive analysis was used and followed the constant comparative method. Four themes emerged from the data including (a) Clinical guidelines and provider discretion influence obesity care; (b) Facilitators and barriers to discussing weight and small step strategies; (c) Informal referrals are made for weight management in community settings; and (d) Opportunities and challenges for integrating clinical and community services for weight management. Facilitating referrals to effective programs, ideally with a feedback loop could coordinate care and enhance accountability, but education, compliance, and cost issues need addressed. Care may be offered but not be well-aligned with clinical guidelines. Knowledge gaps regarding community programs’ offerings and efficacy were evident. Referrals could be systematically promoted, facilitated, and tracked to advance weight management objectives.

## Teaser

Clinicians should provide or refer persons with obesity to treatment but care may not be well-aligned with guidelines and gaps about evidence-based programs were observed.

**Implications**

**Practice**: Clinicians need innovative solutions to make the referral process to weight management in community-based, effective programs more systematic, facilitated, and trackable to advance population health objectives for obesity.

**Policy**: Integration of community and clinical services is a path forward for reducing the proportion of adults with obesity and integration models should be supported with payment models, data sharing, compliance, and education.

**Research**: Future research is needed to examine integration models and the requisite community, clinic, provider, and person-level factors that result in referrals for obesity treatment, program participation, and health outcomes.

## INTRODUCTION

The prevalence of obesity continues an upward trend [1] and is addressed by a variety of clinical guidelines but care is sparse. Obesity is a complex and chronic disease associated with many conditions including cardiovascular disease, type 2 diabetes, and cancer, as well as an increased risk of mortality [2, 3]. Recognition of obesity as a disease by the American Medical Association [4] has not translated into a shift in how health care providers view obesity as indicated by lack of knowledge [5] of evidence-based recommendations for guideline-driven clinical care (offer or refer) [6] and national downward turn in visits for obesity care [7].

Clinical guidelines issued by a host of professional associations and a government-commissioned task force are unified on the message to offer or refer persons with obesity for obesity care. The United States Preventive Services Task Force (USPSTF) [8] recommends that health care providers offer or refer patients with obesity for weight management. The American Academy of Family Physicians endorse the USPSTF guidelines whereas other professional associations issued independent but complementary guidance. The American College of Cardiology/American Heart Association/The Obesity Society (ACC/AHA/TOS) [6], American Association of Clinical Endocrinologists/American College of Endocrinology (AACE) [9], and the American College of Obstetrics and Gynecology (ACOG) [10] all recommend that patients with obesity be offered counseling or referred for treatment. There are nuanced differences between the specialty guidelines. For example, the ACC/AHA/TOS recommends referral to electronically delivered interventions that provide personalized feedback and commercial programs that provide counseling if those programs are supported by scientific evidence of safety and efficacy but the AACE’s recommendation on referral is limited to bariatric surgical programs.

Barriers to offering care have been well-described in the literature but there is limited understanding of the challenges and opportunities for referrals. Generally, barriers to offering obesity care have contributed to a sense of frustration among health care providers who cite low patient motivation, limited reimbursement for obesity-related visits and pharmacological treatment, lack of training, low self-efficacy in lifestyle modification counseling, and high time demands for counseling [11–15]. Less is known about the specific barriers that specialists encounter in offering care, however one study reported that gynecologists are challenged by some of the same issues faced by primary care providers (PCPs) [16]. Looking beyond care offered in the clinic, nearly all clinical guidelines endorse referring persons with obesity to evidence-based intensive behavioral interventions for weight management (ACC/AHA/TOS; USPSTF; ACOG) or to specialty bariatric centers (AACE; ACC/AHA/TOS). However, there is limited evidence about health care providers’ perceptions about referring persons for obesity care with the exception of bariatric surgery [17]. Evidence-based intensive behavioral interventions are available in the community in a variety of modalities (e.g., in-person, virtual, hybrid) and formats (individual, group).

The aim of this study was to evaluate the perspectives of PCPs and specialty providers related to weight management care in clinical settings and referrals for weight management, with the latter emphasizing evidence-based community programs. Given the dramatic increase in the prevalence of adult obesity and downward turn in visits for obesity care, a better understanding of perceptions to making referrals to health services in the community may advance public health goals to reduce obesity (https://health.gov/healthypeople).

## METHODS

### Participants

This was a qualitative study of PCPs and specialists (cardiologists, endocrinologists, gynecologists, and orthopedists) in the USA. The study was approved by the Geisinger Institutional Review Board (2019-0292) and conducted in 2019. A national survey research company (Qualtrics) surveyed an existing panel of PCPs (convenience sample) to identify interest. Next, respondents were telephoned to achieve a purposive sample of approximately 50 PCPs that served rural, urban, and suburban populations, with a goal that providers would be distributed to represent each population type, and who practiced in that setting for at least 1 year. Participants from specialties where weight management was expected to be discussed due to obesity-related comorbidities (cardiology, endocrinology, gynecology, and orthopedics) were similarly recruited to achieve a purposive sample of approximately 32 specialists but there was not an attempt to distribute the specialty providers by population type, mainly because there are few specialists in rural areas. Participant demographic data were collected by Qualtrics, the interviewer, or reported by the participant via curriculum vitae.

### Moderator’s guide

The moderator’s interview guide was developed by the study team and informed by formative interviews with clinicians and thought leaders in the field of obesity practice and science. The interview included questions about familiarity with clinical guidelines, specifically the 2013 ACC/AHA/TOS guidelines [6]; confidence in delivering or making referrals to lifestyle interventions and factors that influence care and referral decisions; confidence in prescribing pharmacological interventions and making referrals for bariatric surgery evaluation; successful and failed conversation starters about weight management; and impressions about the feasibility of making referrals to community-based intensive lifestyle interventions.

### Procedures

Qualtrics scheduled participants with a researcher, who confirmed the appointment by subsequent email. Interview calls opened by confirming the participant’s availability, receipt of study information, answering questions, and obtaining consent. A semi-structured interview strategy was used to encourage conversation. Each interview was conducted by telephone at the convenience of participants, audio-recorded, and transcribed verbatim. The researchers directly involved in data collection (L.B.D., D.J.H.) and one additional researcher (A.M.P.) read and discussed the transcripts as they were produced, and reached consensus about the point of data saturation.

### Analysis

Transcripts were analyzed using thematic analysis, a process that involves six phases including familiarization with the data, generation of initial codes, searching for themes, reviewing themes, naming themes, and producing a final report [18]. Study team members (L.B.D., A.M.P., D.J.H.) read the transcripts and used an open-coding approach to compare initial impressions (e.g., initial codes) from the data. After consensus was reached on initial codes, (L.B.D.) applied an open-coding strategy to code all transcripts. Atlas.ti was used to manage the data. Study team members derived the themes from the data using an inductive, constant comparative strategy through a written review and Socratic discussion of coded data to reach consensus on emergent themes.

## RESULTS

A total of 33 PCPs and 31 specialists participated out of 87 and 72, respectively, who were screened as eligible for participation (Table 1). Participants did not dropout or refuse, per se, as data saturation was reached and there was not a need to continue to schedule interviews. Most PCPs practiced in suburban settings. All PCPs and all but two specialists had practiced for more than 5 years in their current setting; two specialists had practiced for 3–4 years in the current setting. Interviews lasted 12–15 min on average.

**Table 1:** Characteristics of primary care and specialist providers interviewed

	Primary care (*N* = 33) *n*(%)	Specialist (*N* = 31) *n*(%)
Allopathic Medical Doctor (MD)	28 (75)	28 (90)
Osteopathic Medical Doctor (DO)	4 (12)	3 (10)
Certified Nurse Practitioner	1 (3)	–
Cardiology	–	8 (26)
Endocrinology	–	6 (19)
Gynecology	–	11 (35)
Orthopedics	–	6 (19)
Mean age in years (range)	54 (41-65)	62 (57–66)
Rural practice setting	5 (15)	0 (0)
Suburban practice setting	13 (39)	8 (26)
Urban practice setting	7 (21)	21 (68)

Totals may not equal 100 as some providers did not respond to demographic questions.

Four primary themes emerged from the inductive analysis of coded interview data (Table 2). The primary themes include: (a) Clinical guidelines and provider discretion influence obesity care; (b) Facilitators and barriers to discussing weight and small step strategies; (c) Informal referrals are made for weight management in community settings; and (d) Opportunities and challenges for integrating clinical and community services for weight management. Nuanced differences between PCPs and specialists are reported.

**Table 2:** Select quotes from providers about offering care or making referrals for obesity

**Theme 1. Clinical guidelines and provider discretion influence obesity care.**
“I have been aware of such guidelines [ACC/AHA/TOS] since they’ve come out, when they have been modified and all that stuff. So, I am aware of what they would like to do. I’m aware of how it’s applied to clinical practice and I try to fit that in, the parameters in the best I can depending on the patient’s situation. So, it’s not anything new to me. I’ve been referring back to them for years and years and you know you always look to the guidelines.”—Primary Care
“Yes, so to be honest, I don’t really use those guidelines for my obesity weight loss management. I just kind of do my own thing.”—Primary Care “For weight loss? I’m not really that familiar. I didn’t know they had guidelines.”—Primary Care
“American College of Endocrinology, I am familiar with because that is where I belong.”-Endocrinologist “I think guidelines are a start…I am also looking at patient characteristics…for many, I serve as their primary care physician as well. I am looking at the totality of and also how motivated they are…part of the challenge is we need a behavioral solution for obesity, and we do not have one.”—Gynecologist
“…depends on BMI. If the patient is overweight or obese, the treatment is different. You try to go for lifestyle changes…get the help of a nutritionist,…see whether they have additional risk factors or complications like hypertension, MetS [Metabolic Syndrome], diabetes, cardiovascular disease, etc.”—Cardiologist
**Theme 2. Facilitators and barriers to discussing weight and small step strategies.**
“It certainly is something that a lot of times the patient brings up…I would say over 80% of them freely admit that they believe their problems are related to body size.”—Cardiologist “One of the biggest things is they are usually on multiple medications so I tell them that if they lose weight, whether it be through diet and exercise or some type of program, or through bariatric surgery, the likelihood that they will be on fewer medications is important.”—Cardiologist “At first when you talk to them about weight loss, they are kind of resistant. People 200-300 pounds, they don’t really see why they need to lose that much weight. It may be something you would talk to them about maybe the second or third visit.”—Primary Care “Whether it’s obesity, smoking cessation, yeah, we just kind of assess readiness to change and seeing where they’re at in the change process. You know, whether they’re precontemplative or contemplative…whenever I’m asking a patient to make a behavioral change.”—Primary Care “…patients who come to my office wanting a quick fix, not really invested in trying to implement a lot of these strategies, and real basic difficulty with patients understanding what is required of them.”—Primary care “Well, I would say 70% of the reason people are overweight is because of motivation.”—Primary Care “They’ve got to change their lives, they’ve got to make small changes, small long-lasting changes and try to lose a couple of pounds every month and go from there.”—Primary Care “We have them keep a food diary. Half the time they say they are not eating anything and with the food diary, they are eating a lot more than they are saying.”—Primary Care “Well my first intervention would be to discuss with them is exercise.”—Primary Care
**Theme 3. Informal referrals are made for weight management in community settings.**
“I see 40 patients a day. I mention [WW] probably 10 times a day.”-Gynecologist “[The program] puts them in touch with people who understand the way people who are overweight think and eat, and what food and, in some cases, are addicted to food, so it puts them in touch with people who are appreciative of the way they feel and, so I think that’s a good point, and it does start them at least on a path of healthier eating”—Primary Care “I won’t necessarily refer them myself, but I will kind of in conjunction with the primary care doctor, look this guy needs a knee replacement but his BMI is too high, what do you think about bariatrics?” –Orthopedist
**Theme 4. Opportunities and challenges for integrating clinical and community services for weight management.**
“We actually, in our electronic medical records (EMR), have a referral to the YMCA program for prediabetes…we built a direct link so that it actually goes to YMCA. We have been doing it for about a year. It’s worked out pretty good. We are getting folks sent over there.” “[program] has helped to reverse some of the diabetes. I would like to refer you to them. They may be contacting you, do I have your permission to do so?” “Most patients, after I explain it, are willing to at least have a discussion with them...It’s actually coming from the physician, I think it’s powerful.”—Primary Care “If they know that I’m getting information back from [the program] …I can be a cheerleader for them…because right now, when I give a verbal referral, I don’t really follow-up until they come…and it’s just not where it needs to be.”-Gynecologist “Important to see some objective measures if patient is improving or not…adherence…how often attending meetings…following along with plan suggested.”—Cardiologist “If they had success with [the program] and they were doing well and the referral would maybe be more affordable to them, the patient satisfaction would be higher, and they would value that referral.”-Primary Care “…need some kind of representative from [these programs] come and talk to the doctors. Like have some kind of relationship with the doctor…like a drug rep that comes…positive influence to the patient because my doctor recommended.—Endocrinologist “…as long as it is HIPAA compliant and nobody is going to leak any confidential information about the patient, I think it is a great idea.”—Endocrinologist I certainly wouldn’t want to give the impression that I’m trying to sell them something or steer them in a certain direction.”—Primary Care “I can see the patient and have another office visit with them, then referring out is just sort of taking away business from myself, so that would be one of the downsides.”—Primary Care

### Clinical guidelines and provider discretion influence obesity care

Providers were asked about awareness of the ACC/AHA/TOS guidelines [6]. PCPs were familiar with these whereas specialists were not, and this was true even among cardiologists despite the guidelines being co-authored by the ACC. PCPs had a range of instrumental knowledge regarding the guidelines and noted their complexity. Additionally, a few PCPs noted the more recent USPSTF recommendations [8]. Not all PCPs were aware of obesity treatment guidelines and several noted that their experience guides their practice rather than guidelines.

Specialists were more likely to be aware of guidelines offered by their professional board. Specialists discussed obesity management through the lens of the chronic disease that they specialize in, for example, an endocrinologist discussed “diabetics with obesity.” However, gynecologists aimed to deliver a more holistic approach and were the only professionals that identified behavioral change counseling as a gap in care.

### Facilitators and barriers to discussing weight and small step strategies

Providers perceive they talk about weight “all the time” and this discussion is facilitated by relationship building and framing obesity as a health issue. Some providers aimed to develop a patient-provider relationship over a few visits prior to raising weight management. Other providers used a didactic approach to communicate chronic disease and/or the importance of adiposity-related risk indicators to support weight management discussions. A few PCPs used Stages of Change [19] language to describe their practice of assessing patient readiness for weight loss. Providers classified patients in a stage but did not provide details regarding how they support patients in the transition from one stage to another. In contrast to PCPs and gynecologists, other specialists did not discuss assessing readiness and instead, their approach focused on clinical risk factor assessment. The main barrier to discussing weight was the perception that persons with obesity lack motivation. PCPs commonly conveyed an erroneous belief that low patient motivation is the etiology of obesity.

Providers described offering lifestyle modification advice, generally advising small step strategies. Only endocrinologists discussed the use of pharmacotherapy. In terms of small steps, some providers suggest that patients increase their physical activity level as the first step but most providers ask patients to keep a food diary. Providers discussed advising patients of treatment options by using a directive or didactic approach rather than a counseling approach. One PCP mentioned using the inquiry, “What makes the step easier, more difficult for you to implement?” but use of this technique to support behavioral change was rare.

### Informal referrals are made for weight management in community settings

Nearly all providers recommend commercial programs in the community as efficacious options for weight management. Programs such as WW (formerly Weight Watchers), Jenny Craig, Nutrisystem were mentioned as well as the Diabetes Prevention Program (DPP) for those with prediabetes. Providers also refer patients to registered dietitians who are found in traditional (e.g., cardiac rehabilitation, diabetes education programs) and nontraditional settings (grocery stores). Those with registered dietitians in-house or in-network, prioritize this referral resource. PCPs rarely refer to bariatric surgery whereas all specialists, except gynecologists, refer to bariatric surgery.

Infrastructure to facilitate referrals from the clinic to community is available. One provider discussed the ability to refer a person with prediabetes to a DPP program offered in the local YMCA [20] and proposed this as a model that could be extended for obesity treatment.

### Opportunities and challenges for integrating clinical and community services for weight management

Mechanisms to facilitate the referral process and a feedback loop to strengthen the value of the referral were identified as integration opportunities. Patient consent, privacy protections, and provider education and outreach would facilitate referrals. Providers widely endorsed referrals and strategies that would make referrals easier to arrange for the patient, as a PCP noted, “Anything that facilitates contact or access is a good thing. I don’t have a problem with a voucher or direct electronic medical records (EMR).” Patients and providers utilize varied communication tools from in-person to telehealth and written scripts to electronic referrals, and familiarity with the tools would likely influence adoption at the provider and patient levels. Importantly, there is a need for patient consent, to ensure that the patient is aware of and in agreement before he or she is contacted by a community-based program. Data sharing, even with privacy protections, from the provider to the community-based program could be limited to contact information necessary for the referral process. Many providers speculated that outcomes from a demonstration project could influence their adoption of referrals.

Providers noted the need for details about community-based programs and suggested the pharmaceutical industry’s education and outreach approach as a model. Providers speculated that a systematic strategy for referrals could have rippling effects on patient satisfaction and the value of care. After referrals are made, a feedback loop is desired to aid the provider in monitoring progress, offering support, and modifying clinical treatment. Providers speculated that patient knowledge of such a report may boost accountability for engaging in the program and trying strategies. Regular communication, whether monthly or quarterly, could be conveyed from the community weight management program to the provider as an electronic consult report that includes key variables about attendance, behavioral strategies (e.g., meal plans, activity goals), and weight trends from start to goal.

Out-of-pocket cost, program availability, and medical complexity are patient-level barriers that may affect referrals while providers may face a conflict of interest. Although providers indicated that patient request is a major driver of referral for obesity care, out-of-pocket cost for patients was a major concern. The complexities in determining exact costs of the programs are a direct barrier for patients and these complexities function as a barrier to providers recommending community weight management programs. Providers noted the need for sustainable coverage, far beyond a 6-month program, to care for obesity. Insurance coverage emerged as the most common solution for the cost of care and providers endorsed coverage for programs with demonstrated effectiveness. Some providers were concerned about the availability of in-person care but many providers observed the expanding variation of program formats as a facilitator that allows tailoring to patient preference. Formats mentioned were in-person versus telephonic or video, group versus individual, and apps to support behavioral change. Providers were hesitant to refer patients for obesity care when comorbidities are present due to concern that a community program could not adequately address medical complexities. In these situations, providers emphasized that care should be arranged within the clinical system. This point was salient when pharmacotherapy or bariatric surgery were likely to be indicated. Finally, referrals to programs outside of the traditional clinical system may present a conflict of interest for providers. If obesity can be effectively managed in their own clinic, then provider’s business interest is to maintain this model rather than offer referrals. A few providers were concerned that patients may speculate that they would receive a personal benefit for each referral and that an economic incentive would outweigh clinical opinion about the evidence base of a program.

System-level issues such as patient privacy and data security concerns present challenges to sharing patient information, however many felt that such challenges could be resolved. Likewise, EMRs with embedded functionality for data sharing, whether unilateral or bi-directional, are desired by providers over locally derived solutions.

## DISCUSSION

This study demonstrated that primary care and some specialty providers perceive that they discuss obesity with their patients and offer care, but awareness of clinical guidelines is variable. Professional guidelines may influence orientation toward practice as endocrinologists discussed pharmacotherapy, uniquely described in the AACE guidelines, and few orthopedists offered obesity care, perhaps reflecting an absence of obesity guidelines in this specialty. Future research should determine strategies to improve the uptake of guidelines, conversation starters, brief counseling, and identifying when a referral is indicated and where to refer for evidence-based treatment. Providers refer patients with obesity to dietitians and bariatricians but providers’ lack of knowledge about the availability of these obesity specialists and cost limits patient access. Obesity specialists can be located using a “Find a Provider” online directory (Academy of Nutrition and Dietetics, American Board of Obesity Medicine, American Society for Metabolic and Bariatric Surgery). Community-based programs are informally recommended for weight loss, but these are not recognized as referrals, per se, and there is great enthusiasm among providers for a streamlined referral process to effective community-based programs. Optimized implementation should attend to provider awareness, patient access, and agreement, and, if automated, private and secure information exchange. Post-referral feedback from the community-based program to the provider could enhance obesity management with coordinated care and potentially improve patient accountability. Additional research should determine effective strategies to improve referrals from clinical care to evidence-based obesity treatments and the utility of feedback loops, as both are gaps in the literature.

Consistent with proposed standards for clinical providers [21] and practical steps for implementing guidelines [6, 8], most providers assessed and discussed obesity-associated comorbidities. However, no provider specifically acknowledged that obesity is recognized as a chronic disease in and of itself, nor was there mention of the role (or assessment) of genetic background, ethnicity, or social determinants when considering risk associated with body mass index [21]. Notably absent was providers’ use of the recommended practice of asking patients for their permission to discuss weight. Also consistent with guidelines, providers recommended small lifestyle changes to manage obesity, with the default being a food diary. Providers discussed assessing a patient’s readiness to pursue weight management and evaluated readiness according to the Stages of Change model. However, this practice is not described in the guidelines [6, 8] or standards and the evidence is inconsistent regarding the use of this classification system to predict behavioral change or weight loss success [22–24]. Although providers offered lifestyle modification advice, there was little evidence of providers using counseling techniques to understand a patient’s motivation, goals, and barriers to facilitate behavioral change [21, 25]. Providers described using a didactic educational approach to inform patients of risk but patient engagement and participation in decision making were rarely mentioned. Brief education about risk and specific recommendations for the patient to act (food diary) are understandable given time constraints and other visit priorities. Regarding the default strategy, a follow-up visit would be indicated to review eating patterns, but such visits are not routine [26] and even so, still warrant the application of behavioral counseling. Accordingly, the Society of Behavioral Medicine has advised providers to consider referrals to obesity specialists (dietitians, behavioral psychologists) or effective community-based programs to optimize weight loss success [25]. Providers’ lack of training in obesity management, low self-efficacy in lifestyle modification counseling, and high time demand for counseling [11–15], further support the need to explore how referrals can advance obesity management.

A critical element to address with providers is weight bias. This study observed stigmatizing, biased, and offensive statements from providers who failed to recognize obesity as a disease. This finding adds to substantial evidence describing weight bias among health care providers [27, 28]. Providers commonly perceived that patients with obesity lack motivation yet providers rarely conveyed the use of counseling techniques to understand patient motivation or goals. Additionally, we observed that providers use a didactic approach to tell patients about health concerns related to weight. Given that patients with obesity are aware of health risks and have been struggling with weight for years before discussing with a provider [26], a didactic approach is insufficient and potentially harmful [28]. A patient-centered approach is recommended for meaningful and productive conversations about obesity. Strategies like asking patients for permission to discuss their weight and health are useful to engage patients in a conversation rather than a didactic lesson, per se. Providers are advising lifestyle modification and specific self-monitoring strategies but some recognized behavioral change counseling as a gap in their clinical care.

Despite guidelines recommending referral, this is an under-studied area and opportunity to address unmet needs. Herein, clinical and community resources are recognized as settings to receive referrals. Providers discussed their practice of referring patients to clinical services, for example, registered dietitians and obesity specialists, with the former being most commonly mentioned among cardiologists, gynecologists, and endocrinologists and the latter being least commonly mentioned among those in primary care. A low frequency of referrals from PCPs to dietitians and obesity specialists is a common state of affairs [26, 29], in part related to the limited availability of obesity specialists or the providers’ lack of familiarity with these local resources [11, 12]. Enhanced promotion and awareness of existing nutrition and obesity specialists to a variety of provider types may facilitate referrals for obesity care [16].

Community-based programs for weight management including commercial weight loss programs are recommended by all types of providers interviewed. Consistent with the emerging literature [16], providers expressed strong enthusiasm for a more formal system to recognize community referrals as a care pathway, specifically for programs with an established evidence base. One provider described the experience with the Centers for Disease Control and Prevention (CDC)’s DPP program for people with prediabetes, essentially serving as a real-world demonstration model of the feasibility of connecting clinical and community health services [20]. The provider had received information to educate the patient, obtain the patient’s agreement for a referral, and then made a referral through the EMR to a community-based program. The DPP is recognized as a reimbursable program for people with prediabetes by the Centers for Medicare & Medicaid and costs are otherwise covered using a variety of models [20]. Integration of clinic and community health services has been advocated for obesity treatment to better coordinate care and meet population needs to advance public health objectives for healthy weight [30]. The DPP is an emerging example of clinic-community integration and could inform future applications for obesity management. Provider education about commercial programs is warranted to facilitate their ability to tailor referrals to meet patient needs and preferences. At minimum, education should address weight bias, framing obesity as a disease [4], counseling techniques used by commercial programs to facilitate behavioral change [31], formats available including virtual and app options that align with the transformation to telemedicine and digital care models, and program efficacy [31, 32].

Referrals for obesity care present challenges for providers including patient access, the need for providers to obtain patient agreement before referred care is arranged and, if automated in an EMR, information security and privacy concerns. Providers observed variable patient access to clinical and community obesity specialists and while there are efforts advocating for expanded access to manage obesity as a chronic disease [21], the outcomes of participating in community-based weight management programs are similarly beneficial regardless of direct or covered payment [33, 34]. Informing the patient of the anticipated contact and confirming patient permission for contact may be critical steps to implementing referrals to community programs for patient engagement and privacy protection, respectively. PCPs may be uniquely challenged with conflicts of interest if effective care could be offered within the clinic but, like other chronic diseases, a decision to refer patients for more comprehensive management can be indicated.

The practice of referring patients to community programs could be facilitated by improved provider awareness of available programs, their effectiveness, and patient access including covered and direct costs. Providers interviewed were enthusiastic about the ability to directly refer patients to community programs when the outreach and education for such programs were directly offered to providers. This education model is utilized by the pharmaceutical industry, CDC DPP, and is familiar to providers [20, 35]. Independent or collaborative efforts by community-based weight management programs may consider a similar strategy to educate providers of program details and effectiveness. Such strategies may emphasize clinical guidelines and proposed standards as our results showed that provider awareness of clinical guidelines was variable, but an understanding can influence practice [36].

Paper vouchers may be a short-term and feasible strategy for executing referrals whereas system adaptions may be needed to make automated EMR referrals from clinic-to-community settings. Providers described paper vouchers as being similar to a coupon for medications, specifically for those not fully covered by the patient’s insurance. However, providers were most enthusiastic about the ability to automate a referral through an EMR to community-based weight management programs. Providers speculated that such an innovation would fundamentally improve obesity treatment, patient satisfaction, and the value of care. Similar features have increased referrals from clinic-to-community DPP programs [35]. Providers emphasized the need for system changes at the federal level by establishing coverage for obesity as a chronic disease (similar to prediabetes), at the corporate level by establishing data security protections in EMR licensing software to allow for clinic-community data exchange, and at the institutional level by establishing privacy practices that maintain confidentiality within federal policy guidelines. While the approach may seem complex, the CDC DPP program is demonstrating the feasibility of a systems approach [20] and sets a path forward for obesity management.

Providers’ desire for feedback from the community-based weight management program to enhance chronic disease management is a novel finding. Medications may be reduced or adjusted following weight loss or if patients are struggling, there may be a need to adjust medications that contribute to weight gain. Feedback could be modeled on consult reports from specialists providing a brief summary of assessment, treatment, and progress (participation and weight). Report frequency could be quarterly though some providers stated a preference for more or less frequent communication. Importantly, providers speculated that receiving feedback would better enable them to function as a cheerleader, offering supportive encouragement to their patients as they pursue weight management.

The strength of this study is the rich insights from providers regarding perceptions of their practice in offering or referring persons with obesity for care. Additionally, the study included a U.S. sample, five types of providers, and the voices of those who serve rural, urban, and suburban populations. While qualitative methodologies offer deep insight to experiences and perceptions, interviews with busy clinicians were brief and these findings do not represent, nor are they generalizable to, a broader and larger cross-section of the provider population. Additionally, interpretation of qualitative data is subject to bias, although strategies were employed to minimize bias with three reviewers of the transcripts and coded data.

## CONCLUSION

This is the first known study to evaluate the perceptions of specialists in offering care and of a variety of providers regarding their practice in referring patients for obesity management, other than to clinic-based obesity specialists. These findings suggest that providers are routinely recommending community-based weight management programs to their patients despite having variable knowledge about the availability and accessibility of these programs for their patients. Providers desire a greater understanding of community-based weight management program details and effectiveness. Further, providers enthusiastically speculate that systematic strategies to streamline referrals from clinic-to-community with feedback to the referring provider could fundamentally improve obesity care.
